# Achieving long-term water stability and strong exciton–photon coupling in CsPbBr_3_ quantum dots via MOF encapsulation

**DOI:** 10.1515/nanoph-2025-0059

**Published:** 2025-06-18

**Authors:** Chiao-Chih Lin, Shih-Cheng Wan, Cheng-Hui Shen, Zheng-Lin Liao, Yen Liu, Zong Yu Wu, Sheng-Chan Wu, Chia-Kai Lin, Chung-Wei Kung, Hsu-Cheng Hsu, Yu-Hsun Chou

**Affiliations:** Program on Key Materials, Academy of Innovative Semiconductor and Sustainable Manufacturing, 34912 National Cheng Kung University, Tainan 701, Taiwan; Department of Photonics, 34912 National Cheng Kung University, Tainan 701, Taiwan; Department of Chemical Engineering, 34912 National Cheng Kung University, Tainan, 70101, Taiwan; Meta-nanoPhotonics Center, 34912 National Cheng Kung University, Tainan 701, Taiwan

**Keywords:** microcavity, polariton, inorganic perovskite, QD, metal-organic framework

## Abstract

CsPbBr_3_ perovskite quantum dots (QDs) are renowned for their exceptional optical properties, including high quantum efficiency, strong exciton binding energy, and tunable emission wavelengths. However, their practical application is hindered by their inherent susceptibility to environmental degradation. In this study, we introduce a CsPbBr_3_@UiO-66 composite material, where CsPbBr_3_ QDs self-assemble within the microporous framework of UiO-66, a robust metal-organic framework (MOF). This encapsulation strategy significantly enhances the environmental stability of CsPbBr_3_ QDs, maintaining luminescence for over 30 months under ambient conditions and several hours underwater. Temperature-dependent and time resolved photoluminescence (TRPL) measurements further revealed the exciton–phonon interaction within the CsPbBr_3_@UiO-66 material. We distributed CsPbBr_3_@UiO-66 into a hybrid microcavity (MC) and observed strong exciton–polariton coupling, showcasing the remarkable light–matter interaction capabilities of the composite. These findings highlight the potential of CsPbBr_3_@UiO-66 as a robust platform for advanced polaritonic applications, paving the way for next-generation optoelectronic devices and quantum technologies.

## 1 Introduction

Quantum dots (QDs) are pivotal in modern optoelectronics due to their quantum confinement effects, offering tunable emission, high photoluminescence quantum yields, and excellent color purity. These properties have enabled their applications in displays, lasers, sensors, and quantum technologies [1], [2], [3], [4], [5], [6], [7], [8], [9], [10]. Among QDs, lead halide perovskites stand out for their high power conversion efficiency (PCE), superior charge transport, and cost-effective fabrication [11], [12], [13], [14], [15]. Moreover, the versatility of perovskites enables their integration into various optoelectronic components, such as LEDs, lasers, and photodetectors [16], [17], [18], [19], [20], [21], [22], [23], [24], [25], [26], [27], [28]. However, instability against moisture, heat, and light limits their performance and use [29], [30], [31], [32], [33], [34]. This instability degrades perovskite-based devices, limiting their performance and viability. Moisture has been identified as a major factor contributing to perovskite degradation. To address these challenges, template-assisted methods have been explored [35], [36], [37], [38], [39]. Porous materials like alumina, graphene, and MOFs provide spatial confinement, improving stability [40], [41], [42], [43], [44], [45], [46]. MOFs feature tunable internal pores, providing nanoscale spaces to isolate QDs from the environment. MOFs hold great potential among various materials due to their tunable nanoscale pores, effectively isolating QDs from ambient conditions. The pore size and metal nodes of MOFs also provide ideal nucleation sites for perovskite precursors, minimizing aggregation and defects during the growth process [47]. Furthermore, the spatial confinement within MOFs significantly mitigates the influence of external factors such as moisture and oxygen, thereby enhancing both the stability and optical properties of the embedded perovskite. Recent studies have verified the advantages and feasibility of perovskite–MOF systems. For example, Wan et al. reported that amino-functionalized UiO-66(NH_2_) can effectively anchor CsPbBr_3_ QDs, utilizing stable coordination between Pb^2+^ and zirconium nodes to improve photocatalytic performance [48]. Xie et al. demonstrated the *in situ* confined growth of ultra-small CsPbBr_3_ within UiO-66 pores, highlighting how appropriate pore sizes and defect structures suppress non-radiative recombination and enhance luminescence [49]. Additionally, Shi et al. found that embedding MAPbBr_3_ into UiO-66 yields uniformly dispersed QDs that maintain stable emission in encryption and decryption applications, indicating that the MOF framework serves as a physical barrier against moisture and oxygen [50]. MOFs significantly reduce environmental degradation of perovskites and preserves their intrinsic optical performance. In this study, we adopt UiO-66 (Zr_6_(μ_3_-O)_4_(μ_3_-OH)_4_(BDC)_6_, BDC = terephthalate) with missing-linker defects as our template [51]. It exhibits outstanding chemical stability, maintaining a stable structure during the synthesis process and its emission wavelength falls outside the visible light spectrum, thus does not affect the PL signal of the sample. Previous studies have examined CsPbBr_3_ embedded within UiO-66, highlighting its potential for applications such as electrochemical luminescence, photocatalysis, and temperature sensing [48], [49], [50], [51], [52], [53], [54], [55], [56], [57]. Their optical properties, such as exciton-phonon scattering, exciton binding energy, and applications in optoelectronic devices have yet to be fully explored. We examine the stability and optical properties of CsPbBr_3_@UiO-66 through temperature-dependent PL and TRPL and demonstrate strong exciton–polariton coupling in a hybrid microcavity, highlighted by anti-crossing behavior in dispersion curves – an important advancement for polaritonic devices.

## 2 Materials and methods

**Figure 1:**
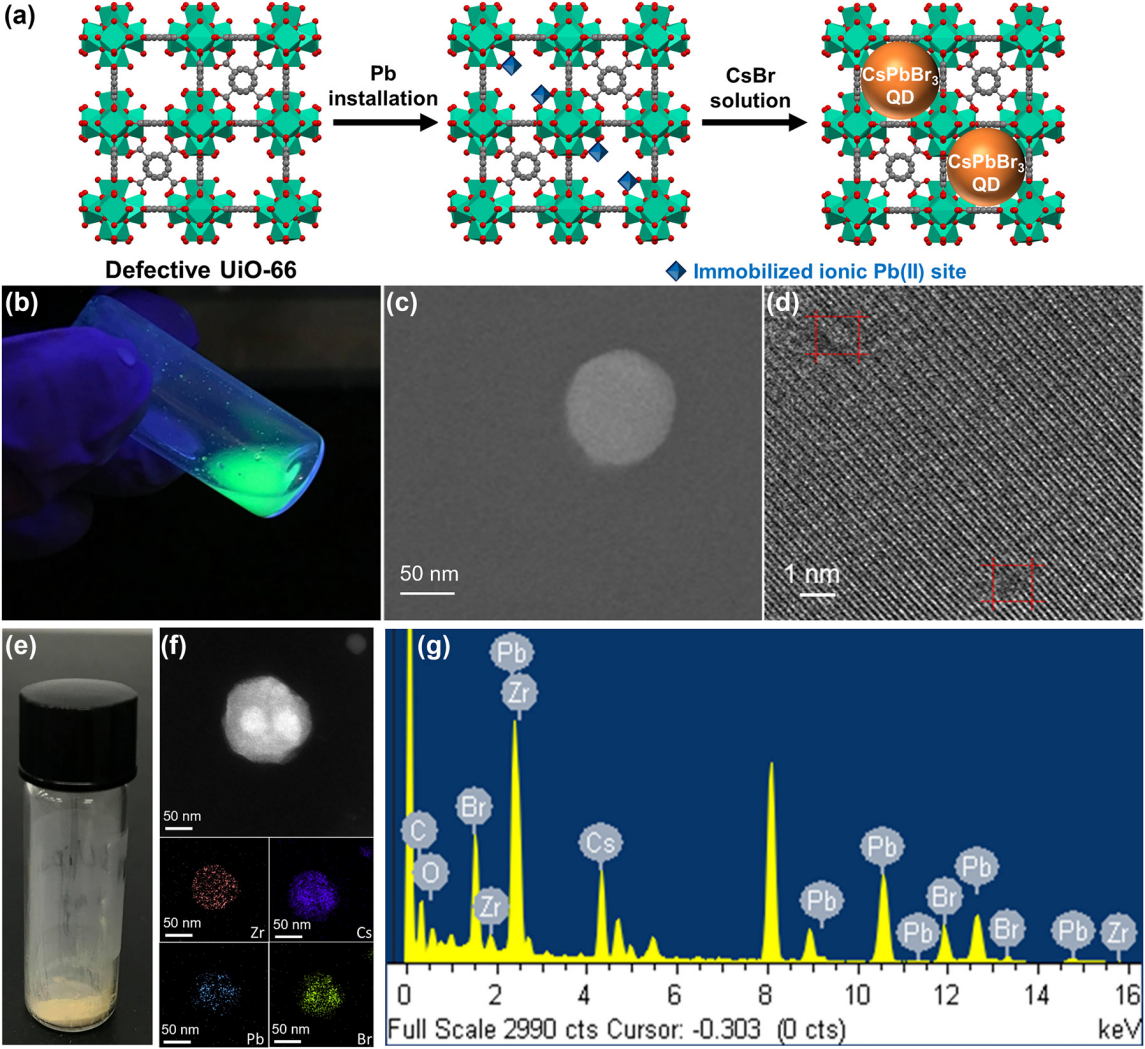
Synthesis and characterization of CsPbBr_3_@UiO-66. (a) Schematic representation of the synthetic procedure for preparing CsPbBr_3_@UiO-66. (b) Photograph of fresh CsPbBr_3_@UiO-66 immersed in water under UV light illumination. (c) SEM and (d) TEM images of CsPbBr_3_@UiO-66. (e) CsPbBr_3_@UiO-66 is stored in vials and preserved in a dry box. (f) HAADF-STEM and (g) EDX elemental mapping images of a typical CsPbBr_3_@UiO-66.

**Figure 2:**
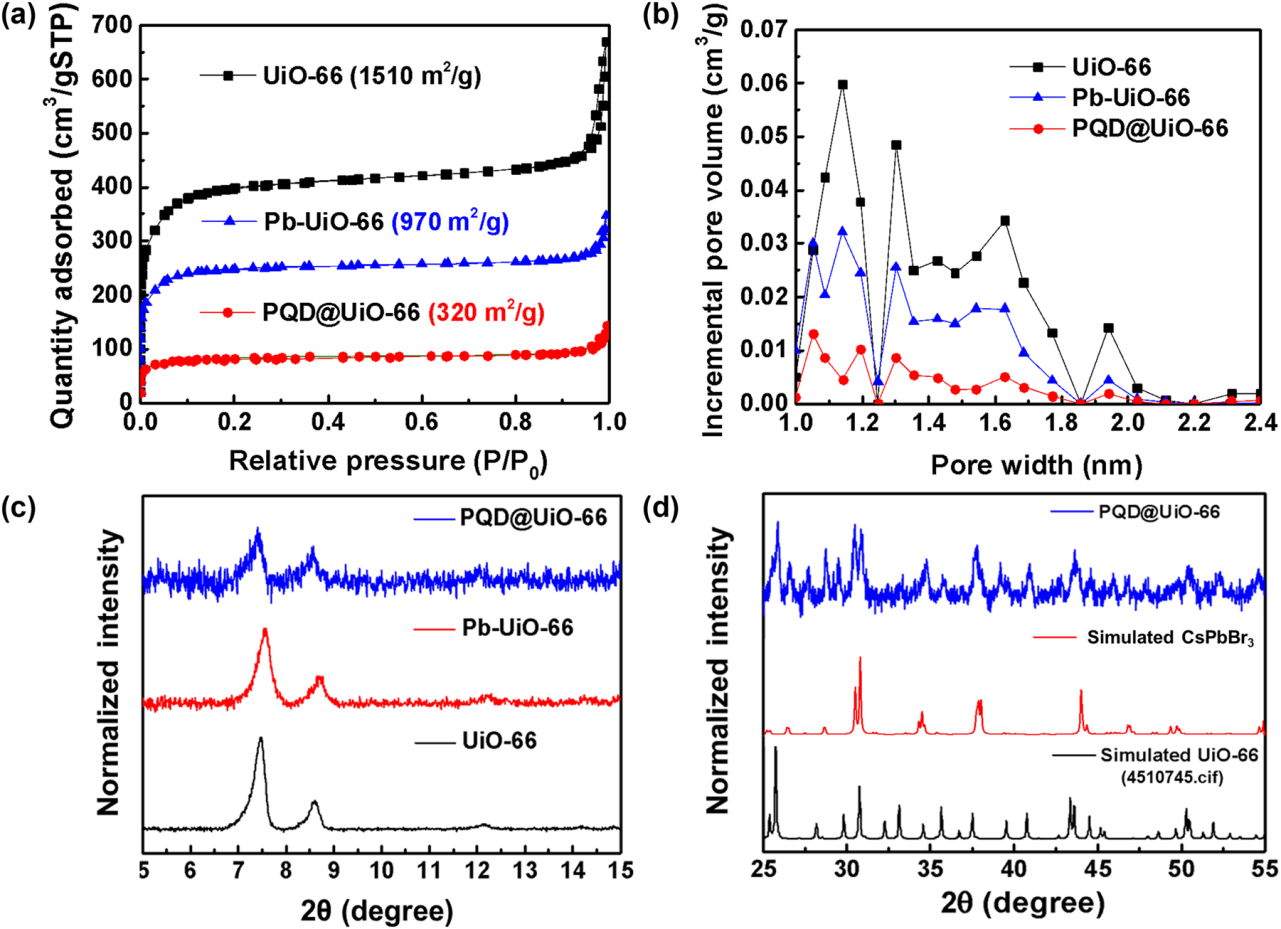
Spectroscopic verification demonstrating the crystallinity and pore size of the CsPbBr_3_@UiO-66. (a) Adsorption isotherm of UiO-66, Pb-UiO-66, and CsPbBr_3_@UiO-66. As the pores are filled, the adsorption quantity decreases, suggesting that the available pore space is limited. (b) Pore size distribution. The synthesized UiO-66 has a pore size of approximately 1∼2 nm, consistent with the TEM observations. (c) Powder XRD spectra of synthesized UiO-66, Pb-UiO-66, and CsPbBr_3_@UiO-66. (d) XRD spectra comparing the experimental results of CsPbBr_3_@UiO-66 with powdered monoclinic CsPbBr_3_ QDs and the simulated results of UiO-66.

The procedures for synthesizing CsPbBr_3_@UiO-66 QDs are depicted in Figure 1(a). UiO-66 powder with missing-linker defects was first synthesized by following the reported procedure [58]. Thereafter, the MOF was subjected to a self-limiting solvothermal deposition in MOF (SIM) method to coordinate spatially dispersed Pb^2+^ ions on hexa-zirconium nodes of UiO-66 [58], [59], [60], [61], resulting in Pb-UiO-66 powder. It is worth mentioning that during the SIM process, transition metal ions such as Pb^2+^ ions were coordinated on the hexa-zirconium cluster of the MOF where the terminal –OH/OH_2_ pairs were initially located, by forming the metal-oxygen bonds between the guest metal ion and the cluster; the formation of such structures has been confirmed both computationally and experimentally for a range of guest metal ions [59], [60], [61]. A CsBr precursor solution was subsequently added to the Pb-UiO-66 powder mixture. After this procedure, a solid with obvious photoluminescent property under the irradiation of a handheld UV lamp was obtained, implying the formation of bulk CsPbBr_3_ QDs within the MOF. This observation also indicates that the Pb–O bonds in Pb-UiO-66 were broken upon the exposure to the CsBr precursor so that the perovskite, CsPbBr_3_, could be generated; the similar two-step formation of pore-confined nanoparticles in Zr-based MOFs has been reported previously [62], [63]. Figure 1(b) shows the CsPbBr_3_@UiO-66 power immersed in water, emitting strong green light for over 180 min of storage, and the accelerated aging test is detailed in Figure S1, Supporting Information. Scanning electron microscope (SEM) micrographs (Figure 1(c)) show that the UiO-66 particles exhibit a uniform morphology with an average diameter of approximately 100 nm. Transmission electron microscopy (TEM) micrographs (Figure 1(d)) reveal the presence of CsPbBr_3_ quantum dots (QDs) within the pores of UiO-66. The high-resolution TEM image shows distinct lattice fringes with an interplanar spacing of approximately 0.58 nm, which corresponds to the (100) plane of CsPbBr_3_, as supported by the X-ray diffraction (XRD) pattern in Figure 2(d). The CsPbBr_3_ QDs in TEM images appear larger (beyond 1–2 nm) due to aggregation or overlap within UiO-66. While the UiO-66 pores provide spatial confinement, localized assembly of multiple QDs can result in the apparent size increase. Additional TEM images presented in Table S1 further confirm the crystallinity of the CsPbBr_3_ QDs, showing consistent lattice plane indexing and interplanar spacing. The observed aggregation does not compromise the framework integrity, as evidenced by the consistent lattice fringes and the uniform distribution of Zr in the framework. These results collectively confirm the successful synthesis of CsPbBr_3_ QDs within UiO-66, maintaining both crystallinity and structural integrity. For the convenience of subsequent measurements and experiments, the remaining CsPbBr_3_@UiO-66 is stored in vials, as shown in Figure 1(e). High-angle annular dark-field scanning transmission electron microscope (HAADF-STEM) and energy-dispersive X-ray (EDX) element mapping images of a typical CsPbBr_3_@UiO-66 are shown in Figure 1(f) and (g). The HAADF-STEM images provide visual confirmation of the spatial confinement of CsPbBr_3_ QDs within the UiO-66 framework. The contrast between the CsPbBr_3_ QDs and the UiO-66 framework demonstrates that the QDs are well-dispersed within the MOF pores, maintaining their dispersion within the framework. EDX element mapping (Figure 1(g)) complements the HAADF-STEM observations by providing quantitative compositional analysis. The EDX mapping shows the uniform distribution of Cs, Pb, and Br elements within the UiO-66 framework, further validating the successful embedding of CsPbBr_3_ QDs. The consistent presence of Zr across the framework confirms the structural integrity of UiO-66 during the synthetic process. The combination of HAADF-STEM and EDX mapping thus demonstrates the spatial confinement of CsPbBr_3_ QDs and the uniform incorporation of the elements, ensuring the stability and integrity of the composite material.

Understanding the changes in porosity during the sequential incorporation of Pb^2+^ ions and CsPbBr_3_ QDs into the UiO-66 framework is essential. If confirms the successful confinement of QDs and evaluating the structural integrity of the MOF. To evaluate the structural integrity and porosity of the synthesized materials, nitrogen adsorption-desorption measurements were performed at 77 K to investigate the porosity of each material. From the isotherm in Figure 2(a), it can be inferred that UiO-66 belongs to the category of typical microporous materials for both Pb-UiO-66 and PQD@UiO-66, exhibiting Type I isotherms [54]. At low relative pressures, the adsorption quantity increases rapidly; as the relative pressure increases, the micropores become fully filled, approaching near-saturation adsorption capacity and resulting in a flat curve. During the experimental process, UiO-66 is sequentially incorporated with Pb and CsPbBr_3_. Due to the larger volume of Pb atoms and the crystallization of CsPbBr_3_ within the pores, the volume of the micropores becomes occupied, leading to a decrease in adsorption capacity. Brunauer–Emmett–Teller (BET) surface area of UiO-66 estimated from the isotherm is 1,510 m^2^/g, similar to that reported previously for defective UiO-66 [64]. After the incorporation of Pb^2+^ ions and the formation of pore-confined CsPbBr_3_, the BET surface areas decrease to 970 m^2^/g and 320 m^2^/g, respectively. Figure 2(b) presents the pore size distributions extracted from Figure 2(a) with the use of the linear density functional theory (DFT) and a slit-pore model. The result shows that the pore size of UiO-66 is distributed in the range of approximately 1–2 nm, originating from both the structurally defined pore of UiO-66 with the size of around 1.2 nm and defective cavities with sizes ranging from 1.4 to 2.0 nm [58], [61]. The pore size of UiO-66 observed here aligns well with the diameter of representative CsPbBr_3_ QD found in the TEM image of CsPbBr_3_@UiO-66 (Figure 1(d)), confirming that we have successfully synthesized QDs and incorporated them into the pores of UiO-66, effectively filling the pores. We next sought to investigate the crystallinity of both CsPbBr_3_ and UiO-66. XRD measurements were performed on UiO-66, Pb-UiO-66, and CsPbBr_3_@UiO-66 to analyze their respective diffraction patterns and evaluate how the incorporation of QDs impacts the framework’s structural characteristics. As shown in Figure 2(c), the characteristic low-angle diffraction peaks of UiO-66 are present in all three XRD patterns, confirming that the overall structure of UiO-66 is retained after the SIM process and the subsequent formation of pore-confined CsPbBr_3_ QDs. It is noted that the intensity of UiO-66 diffraction peaks decreases in the CsPbBr_3_@UiO-66 pattern compared to pure UiO-66. This reduction in intensity does not indicate the destruction of the UiO-66 framework but rather reflects changes in the material’s composition and the reduced relative content of UiO-66 in the composite. The decrease in peak intensity can be attributed to the incorporation of CsPbBr_3_ QDs into the UiO-66 framework, which partially occupies the pores of the MOF and reduces its overall proportion in the composite sample. This observation is also corroborated by nitrogen adsorption–desorption measurements, which show that the BET surface area of the composite remains approximately 320 m^2^/g following the formation of pore-confined CsPbBr_3_. Given that QDs contribute minimally to the surface area, this result strongly supports the conclusion that the UiO-66 framework retains both its structural integrity and porosity throughout the synthesis process. Therefore, while the XRD peak intensity decreases, this change is consistent with the expected compositional shift due to the incorporation of CsPbBr_3_ QDs and does not reflect collapse or destruction of the UiO-66 framework. The combined XRD and BET results provide strong evidence that the structural integrity of the UiO-66 framework is maintained during and after the pore-confined synthesis of CsPbBr_3_ QDs. To confirm the crystallinity of CsPbBr_3_ synthesized within UiO-66, we employed an indirect approach by comparing experimental XRD patterns with simulated lattice arrangements of UiO-66 and orthorhombic CsPbBr_3_ QDs. We found that the CsPbBr_3_ can crystallize within UiO-66 while maintaining a crystalline structure similar to that of CsPbBr_3_ QDs (COD code 4510745). The characteristic peaks located at 26.4°, 26.5°, 27.46°, 28.62°, 30.43°, 30.74° correspond to the (220), (022), (131), (221), (040) and (202) planes. The interplanar spacing calculated from XRD is consistent with the lattice fringes observed in the TEM micrographs. Detailed interplanar spacings for these planes are presented in Table S1 of the Supporting Information. From these interplanar spacing values and their corresponding planes, we derived the lattice constants of CsPbBr_3_@UiO-66 as *a* = 8.2437 Å, *b* = 11.7405 Å, and *c* = 8.1982 Å, demonstrating the preservation of crystallinity and structural integrity in the synthesized composite material.

FTIR spectra of UiO-66, Pb-UiO-66 and CsPbBr_3_@UiO-66 were collected and plotted in Figure S2. Three major characteristic peaks of UiO-66, including those associated with the asymmetric vibration of O–C–O in coordinated carboxylate groups at 1,582 cm^−1^, the C=C in the aromatic ring of the linker at 1,506 cm^−1^ and the symmetric vibration of O–C–O at 1,395 cm^−1^, can be clearly observed in all spectra. These features agree well with those of defective UiO-66 reported previously [65], [66]. Only a tiny peak located at around 1,700 cm^−1^, attributed to the uncoordinated carboxylic groups of linkers, can be observed in the FTIR spectrum of UiO-66, again confirming the successful synthesis of the MOF. The intensity of this peak is almost the same in both spectra of Pb-UiO-66 and CsPbBr_3_@UiO-66, which implies that the installation of Pb^2+^ ions and the further formation of pore-confined perovskite QDs did not significantly alter the coordination of the porous framework. As revealed by FTIR data, no chemical bonds were formed between the organic moieties of MOF and perovskite, suggesting that the CsPbBr_3_ QDs were physically confined within the MOF pore.

## 3 Results and discussion

**Figure 3:**
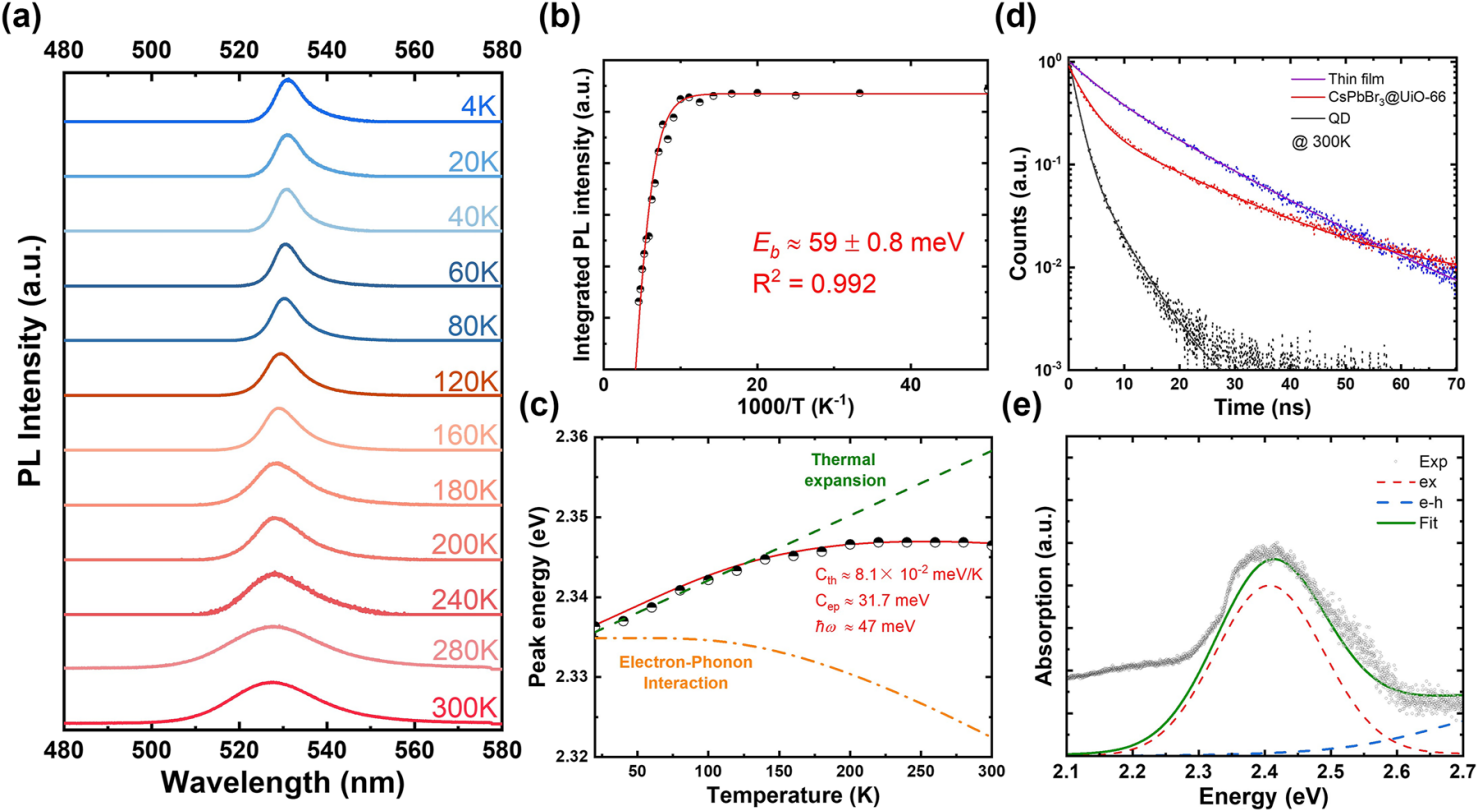
Temperature-dependent PL characteristics of CsPbBr_3_@UiO-66. (a) Temperature-dependent PL spectrum of CsPbBr_3_@UiO-66 measured across a temperature range from 4 K to 300 K. The spectrum exhibits distinct temperature-dependent behavior, where the emission peak progressively shifts towards shorter wavelengths as the temperature increases, accompanied by a broadening of the FWHM. This blue-shifting phenomenon is indicative of the reduction in exciton binding energy with rising thermal energy and is further analyzed in 3(c). (b) Integrated PL intensity plotted as a function of temperature. The data closely follow an Arrhenius model, accounting for the exciton binding energy, as described by Equation (1). (c) Temperature-dependent blueshift of the PL emission peak energy, fitted using the one-oscillator model (Equation (2)). The shift in emission energy is primarily attributed to thermal expansion effects at lower temperatures (<150 K), while electron-phonon interactions dominate at higher temperatures (>150 K). The fitting results, shown by the solid curve, highlight the contribution of thermal lattice expansion (*C*_*th*_) and electron–phonon coupling (*C*_*ep*_), with the observed blueshift further corroborating the excellent thermal stability of the CsPbBr_3_@UiO-66 system. (d) TRPL measurement of QDs, CsPbBr_3_@UiO-66 and TF. The results reveal that CsPbBr_3_@UiO-66 exhibits *τ*_*r*_ comparable to QDs, yet its *τ*_*nr*_ is significantly longer than that of TF. This highlights the dual role of the UiO-66 framework in preserving radiative properties while effectively suppressing non-radiative recombination through reduced bulk defects and enhanced surface passivation. (e) Absorption spectra of CsPbBr_3_@UiO-66, with the experimental data and the theoretical fit. The *E*_*b*_ measured from this spectrum is similar with the value derived from Arrhenius model in Figure 3(b).

Temperature-dependent PL spectra of CsPbBr_3_@UiO-66 were measured from 4 K to 300 K (Figure 3(a)). The integrated PL intensity was analyzed using an Arrhenius equation (Equation (1)):

(1) $$I\left(T\right)=\frac{I_{0}}{1+A\cdot \exp\left(-\frac{E_{b}}{k_{B}T}\right)}$$

where *I*_0_ is the PL spectrum area at 0 K, *E*_*b*_ (meV) is the exciton binding energy, *T* (K) is temperature. *A* is fitting parameter. The fitting results yield *E*_*b*_ ≈ 59 ± 0.8 meV, higher than values reported for bulk CsPbBr_3_ and thin films (Table S5). The exciton binding energy depends on the spatial confinement of charge carriers. In bulk materials or thin films, the continuous density of states (DOS) and spatial delocalization weaken the Coulomb interaction between electrons and holes. In contrast, the nanoscopic pores (∼1–2 nm) of UiO-66 confine CsPbBr_3_ QDs, limiting carrier delocalization and increasing spatial overlap, which enhances electron-hole interaction and raises the energy required to dissociate excitons into free carriers. Unlike bulk and thin-film perovskites, CsPbBr_3_@UiO-66 exhibited a slight blue shift in PL peak positions with temperature increase. This indicates CsPbBr_3_@UiO-66’s thermal and structural dynamics differ from conventional perovskites. To further investigate the underlying mechanisms, we used a one-oscillator model (Equation (2)) to analyze the temperature dependence of the PL peak energy:

(2) $$E_{g}\left(T\right)=E_{g,0}+C_{th}\cdot T+C_{ep}\left(1+\frac{2}{\exp\left(\frac{\hbar \omega }{k_{B}T}\right)-1}\right)$$

where *E*_*g*_ (eV) is the bandgap, *E*_*g*,0_ (eV) is the bandgap without thermal and phonon contributions, *C*_*th*_ (eV/K) is the coefficient of thermal expansion, *C*_*ep*_ (eV) is the coefficient of electron-phonon interaction and *ℏω* (eV) is the average optical phonon energy. The details of the fitting results refer to Table S3. In the low-temperature range (4–150 K), the PL peak energy shows a linear increase, which is well described by the thermal expansion term. The extracted coefficient *C*_*th*_ = $8.1\times 10^{-2}\;\left(\text{meV}/\text{K}\right)$ is significantly smaller than that typically reported for CsPbBr_3_ [64], [67], [68], suggesting that the thermal effects on lattice expansion are suppressed by the confinement within the UiO-66 framework. At higher temperatures (*T* > 150 K), the slope of the PL peak energy curve begins to gradually decrease. This behavior arises from thermally activated electron–phonon interactions, which lead to bandgap renormalization (orange dash line). The fitting yields a negative electron–phonon coupling coefficient $C_{e\;p}=31.7\;\left(\text{meV}/\text{K}\right)$ , indicating that electron–phonon interactions reduce the bandgap energy as the thermal energy increases. The fitted average phonon energy *ℏω* ≈ 47 ± 2 meV indicates that the porous UiO-66 structure amplifies this effect by introducing surface/interface-induced defects, intensifying phonon–electron coupling.

To quantitatively analyze the temperature-dependent PL linewidth broadening, we fitted the measured full width at half maximum (FWHM) using the independent Boson model (3) [69], [70]:

(3) $$\Gamma \left(T\right)=\Gamma_{0}+\frac{\Gamma_{\text{LO}}}{e^{\left(\frac{\hbar \omega_{\text{LO}}}{k_{B}T}\right)}-1}$$

where Γ (meV) is linewidth, Γ_0_ (meV) is inhomogeneous broadening, *ℏω*_LO_ (meV) is longitudinal optical (LO) phonon energy and Γ_LO_ (meV) is the contribution of electron–optical phonon interaction. The details of the fitting results refer to Table S4 and Figure S3. The LO phonon energy extracted from the linewidth analysis is *ℏω*_LO_ ≈ 29 ± 3, whereas the average phonon energy obtained from Equation (2) yields *ℏω* ≈ 47 ± 2 meV. This difference arises because the average phonon energy *ℏω* is an effective value encompassing contributions from multiple phonon modes and electron–phonon interactions across the entire electron population. The clear identification of a dominant LO phonon mode from the independent Boson model suggests that electron–phonon coupling in our CsPbBr_3_@UiO-66 system is predominantly mediated through LO phonons. This finding indicates that Fröhlich-type coupling to LO phonons – rather than acoustic phonon scattering – serves as the principal mechanism underlying the homogeneous PL linewidth broadening in this hybrid material. Such behavior aligns well with previous studies on bulk lead-halide perovskites, where Fröhlich interactions involving polar LO phonons have consistently been identified as the dominant source of PL line broadening at room temperature [71], [72]. The associated Fröhlich scattering rate (1/*τ*_*fr*_) can be expressed as proportional to [73], [74]:

(4) $$\frac{1}{\tau_{fr}}\propto {\left(\frac{1}{\varepsilon \left(\infty \right)}-\frac{1}{\varepsilon \left(0\right)}\right)}^{2}\cdot \frac{1}{\hbar \omega_{\text{LO}}}\cdot \left(N_{\text{LO}}\left(T\right)+1\right)$$

where $\varepsilon \left(\infty \right)$ and $\varepsilon \left(0\right)$ are the high-frequency and low-frequency dielectric constants of the medium and $N_{\text{LO}}\left(T\right)$ is the LO phonon occupation number described by the Bose–Einstein distribution: $N_{\text{LO}}\left(T\right)=\frac{1}{e^{\left(\frac{\hbar \omega_{\text{LO}}}{k_{B}T}\right)}-1}$ . As the temperature increases, the LO phonon population $N_{\text{LO}}\left(T\right)$ rises, enhancing the Fröhlich scattering rate. This intensified electron–phonon scattering at higher temperatures accelerates exciton energy redistribution and momentum relaxation processes, thereby leading to pronounced PL linewidth broadening. The observed temperature-dependent increase in linewidth clearly reflects the thermal broadening mechanisms driven by enhanced electron–phonon coupling.

To examine carrier dynamics influenced by the UiO-66 framework, we performed TRPL measurements and compared them with those of CsPbBr_3_ thin films (TF) and QDs (Figure 3(d)). PL decay curves were precisely fitted using a double-exponential decay function: $I\left(t\right)=A_{1}e^{-t/\tau_{r}}+A_{2}e^{-t/\tau_{nr}}$ , where *A*_1_ and *A*_2_ are the amplitudes of the two exponential decay components. The extracted radiative (*τ*_*r*_) and non-radiative lifetimes (*τ*_*nr*_) for CsPbBr_3_@UiO-66, TF, and QDs are summarized in Table 1 and the average lifetime associated with *τ*_*r*_ and *τ*_*nr*_ can be described as Equation (5) [75]:

(5) $$\tau_{\mathrm{avg}}=\frac{A_{1}{\tau_{r}}^{2}+A_{2}{\tau_{nr}}^{2}}{A_{1}\tau_{r}+A_{2}\tau_{nr}}$$

**Table 1:** Lifetime of QDs, CsPbBr_3_@UiO-66 and TF.

	QDs	CsPbBr_3_@UiO-66	TF
$\tau_{r}\left(\text{ns}\right)$	1.34	3.00	6.35
$\tau_{nr}\left(\text{ns}\right)$	4.04	16.52	15.21
$\tau_{\mathrm{avg}}\left(\text{ns}\right)$	2.50	11.43	13.04

Detailed fitting results of the TRPL measurements for these materials are provided in Table S5 and Figure S4 of the Supporting Information. TRPL measurements of CsPbBr_3_@UiO-66 reveal distinct recombination behaviors, with radiative recombination lifetimes intermediate between TF and QDs, but with longer non-radiative recombination times.

**Figure 4:**
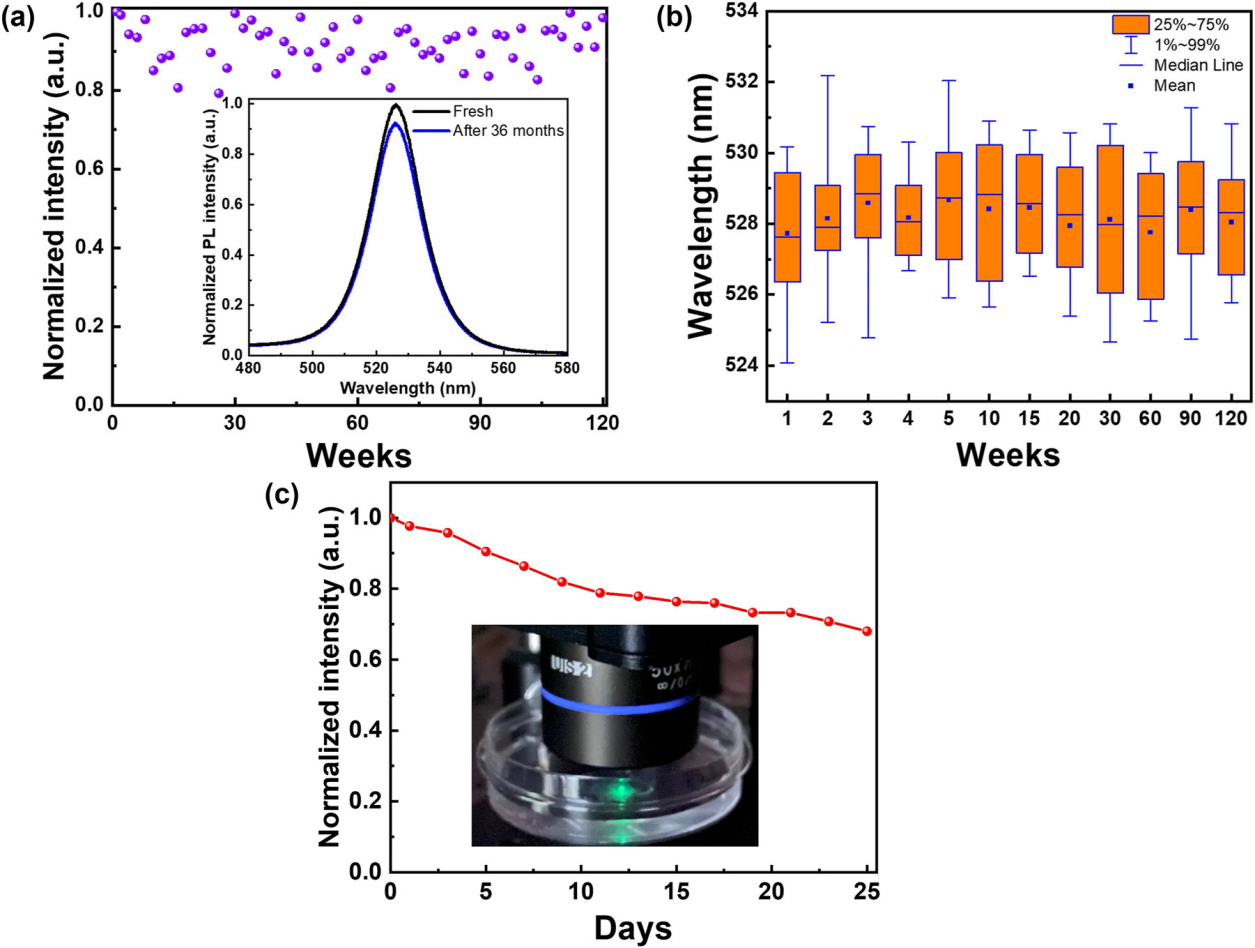
Environmental stability of CsPbBr_3_@UiO-66 in aqueous and ambient conditions. (a) Long-term stability of CsPbBr_3_@UiO-66 in ambient conditions (60 % relative humidity, 25 °C). The normalized PL intensity remains unchanged over 120 weeks, demonstrating the material’s remarkable environmental resistance. Additionally, after being stored in a dry box for 36 months, the PL intensity continues to be stable with no significant degradation, indicating superior long-term stability under both ambient and controlled conditions. (b) Long-term stability of CsPbBr_3_@UiO-66 in ambient conditions (60 % relative humidity, 25 °C). The material exhibits outstanding stability, with the normalized PL intensity remaining constant over a period of 120 weeks, with no signs of degradation even after 36 months in a dry storage environment. (c) Water stability test of CsPbBr_3_@UiO-66. The normalized PL intensity is measured after submersion in water for over 20 days, retaining 70 % of its initial intensity, demonstrating significant resistance to water-induced degradation. The inset shows a photograph of the sample in its aqueous environment, further highlighting its stability in real-world conditions.

In perovskite materials, non-radiative recombination pathways typically involve bulk defects, such as vacancies and interstitials, as well as surface traps caused by surface irregularities [76], [77], [78], [79]. Under low pump fluence conditions, we employed a set of differential equations (Supporting Information) to describe the photogenerated carrier density (*n*_*c*_(*t*)) following PL excitation [80]. This model identifies the presence of two distinct types of trap states in CsPbBr_3_@UiO-66: bulk traps, characterized by fast trapping times, and surface traps, which exhibit slower trapping dynamics [81], [82], [83]. The bulk trap density is ${n_{TP}}^{F}∼1\times 10^{14}$ whereas the surface trap density is ${n_{TP}}^{S}∼6.4\times 10^{18}$ . The bulk trap density of CsPbBr_3_@UiO-66 is lower than that of QDs and TF, whereas its surface trap density is significantly higher than both. We propose that the UiO-66 framework influences non-radiative recombination through a dual role. It acts as a protective barrier for CsPbBr_3_, shielding it from environmental factors like moisture and oxygen, thereby reducing internal crystal defects and passivating bulk traps. Simultaneously, the high surface area and porous structure of UiO-66 create numerous interfaces between CsPbBr_3_ QDs and the framework, which become hotspots for trap states [84], [85]. Additionally, vibrational modes at these interfaces may couple with the electronic states of CsPbBr_3_, introducing further non-radiative recombination pathways. This dual influence highlights the interplay between the structural protection offered by UiO-66 and the trap state formation induced at its interfaces. We also measure the absorption spectrum of CsPbBr_3_@UiO66 at room temperature, and model that in the framework of the Elliot’s theory including hydrogen-like excitonic effects [86], [87], [88]. As shown in Figure 3(e), the absorption spectrum of CsPbBr_3_@UiO-66 clearly reveals an excitonic peak distinct from the continuum band absorption. The exciton binding energy obtained from the absorption spectra in Figure 3(e) is consistent with the value derived from the Arrhenius equation in Figure 3(b). CsPbBr_3_@UiO-66 yields an exciton binding energy *E*_*b*_ ≈ 60 ± 3 meV, which is larger than that of QDs and TF (*E*_*b*_ ≈ 45 ± 5 and 30 ± 4 meV, Table S7). The spectrum’s broad linewidth is attributed to inelastic scattering caused by material defects and phonon interactions. Defects, such as surface traps and bulk vacancies, create localized energy states within the bandgap, broadening absorption pathways. Additionally, strong electron–phonon coupling in the confined CsPbBr_3_@UiO-66 system intensifies these effects, as phonon-induced lattice vibrations further disrupt electronic states, resulting in the observed broadening of excitonic and continuum absorption features. To assess the air and water stability of CsPbBr_3_@UiO-66, it was spin-coated onto a sapphire substrate, and the powder was measured individually. Using a 405 nm CW laser at 1 kW/cm^2^, the normalized PL intensity remained stable under ambient conditions (60 % relative humidity, 25 °C) for over 120 weeks and for 36 months when stored in a dry box (Figure 4(a)). Statistical analysis of the first five weeks showed a median emission wavelength of ∼528 nm (Figure 4(b)), which informs the design of the resonant cavity length model. Additionally, CsPbBr_3_@UiO-66 retained 70 % of its initial PL intensity after 20 days of water immersion (Figure 4(c)).

**Figure 5:**
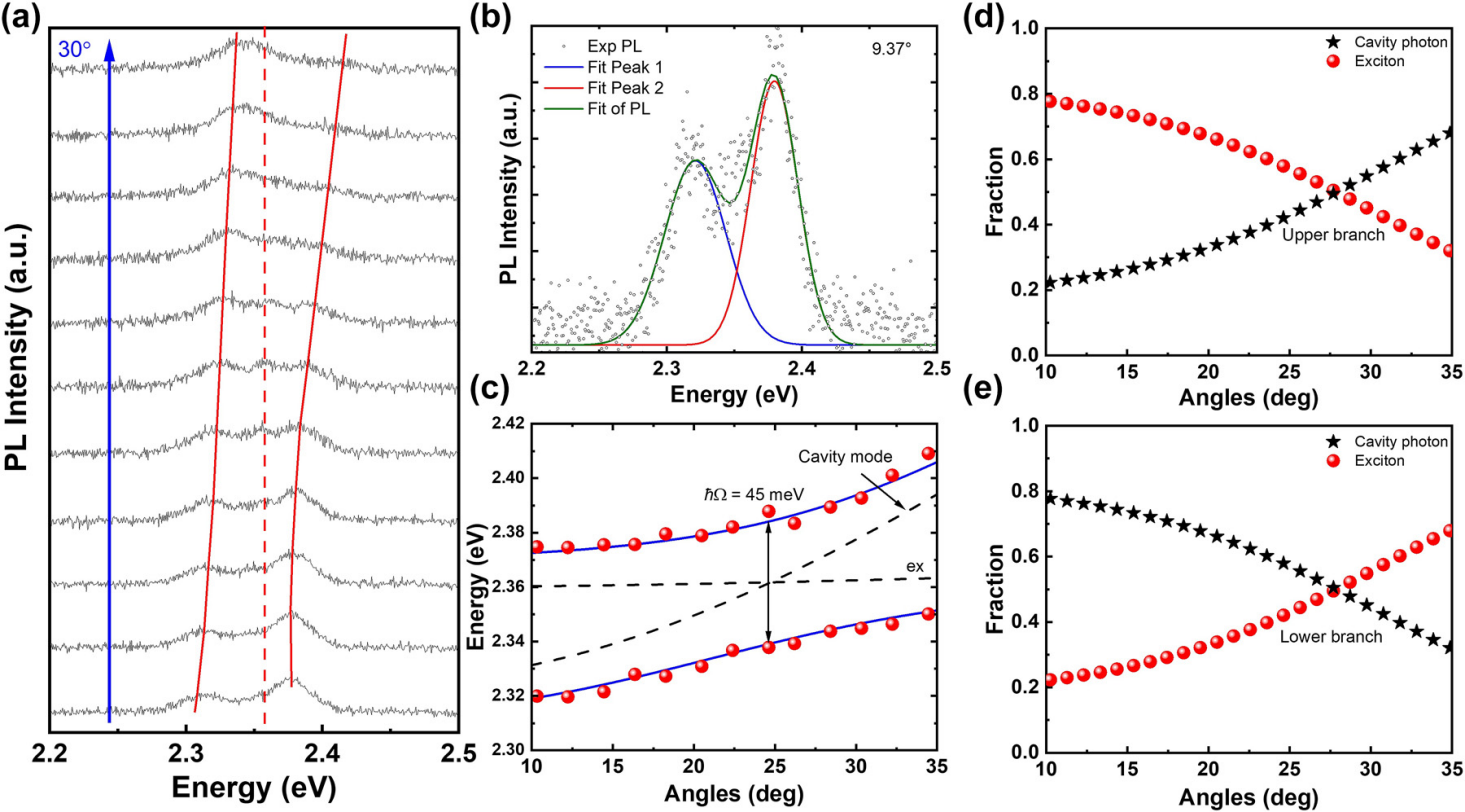
ARPL mapping and polariton dispersion in CsPbBr_3_@UiO-66 microcavity. (a) ARPL spectra for the device from 1.45° to 30°. The vertical red dashed line represents the CsPbBr_3_@UiO-66 exciton energy and the red curves trace the dispersion of microcavity polariton modes. (b) Expanded view of PL spectra features at 9.37°. A significant LPB PL peak is observed around 2.371 eV, as well as a prominent UPB peak at 2.317 eV. The PL spectrum is also fitted to multiple Lorentzian peaks to locate the exact PL peak positions. (c) The energy versus angle dispersion, extracted from the ARPL spectra. The dispersion is fitted to a coupled oscillator model, showing Rabi splitting of ∼45 meV. (d), (e) The corresponding exciton and cavity photon weights of the UPB and LPB dispersions as a function of the angles.

To explore strong light–matter interactions, CsPbBr_3_@UiO-66 was integrated into a hybrid MC with a distributed Bragg reflector (DBR) at the bottom and an Ag layer on top. The cavity quality factor was measured as *Q* = 151. The linewidth of the exciton transition is *ℏ*Γ_*ex*_ = 69.4 meV which is extracted from the PL spectra, as shown in Figure S7(a) and (b). The strong coupling between the cavity modes and excitons in CsPbBr_3_@UiO-66 is investigated by measuring the angle-resolved photoluminescence (ARPL) spectroscopy in Figure 5(a). The PL spectra reveal the formation of polaritonic states, with distinct lower polariton branches (LPB) and upper polariton branches (UPB). Figure 5(b) shows the PL spectrum measured at a specific angle (9.37°), where two emission peaks corresponding to the LPB and UPB are clearly observed. The peak positions were precisely determined using Lorentzian fitting. The results indicate a clear energy splitting between the polaritonic branches. We reach the strong coupling regime at room temperature where the cavity detuning is = *E*_*C*_ − *E*_*ex*_ = −35 meV. To directly investigate exciton–photon coupling, the dispersion curve was fitted using the coupled oscillator mode [89]:

(6) $$\left[\begin{array}{cc} E_{C}\left(\theta \right) & g_{A}\\ g_{A} & E_{ex} \end{array}\right]\left[\begin{array}{c} C\\ X \end{array}\right]=E\left[\begin{array}{c} C\\ X \end{array}\right]$$

where *E*_*C*_ and *E*_*ex*_ correspond to energy levels of cavity modes and excitons coupled by the interaction potential *g*_*A*_, respectively. *E* indicates the eigenvalues corresponding to the energies of polariton modes. The photon and exciton fraction in each LPB and UPB are given by the amplitude squared of *C* and *X*, where |*C*|^2^ + |*X*|^2^ = 1. They are referred to as the Hopfield coefficients. *g*_*A*_ is the exciton–photon coupling strength.

The eigenvalues are given by

(7) $$\begin{array}{ll} E_{\text{UPB,LPB}}\left(\theta \right) & =\frac{1}{2}\left[E_{C}\left(\theta \right)+E_{ex}\right.\\ & \;\pm \left.\sqrt{\left(4{g_{A}}^{2}+{\left(E_{C}\left(\theta \right)-E_{ex}\right)}^{2}\right)}\right] \end{array}$$

From Equation (7), two hybrid modes *E*_UPB_ and *E*_LPB_, are observed. The ARPL data in Figure 5(a) were analyzed, and the fitted peak positions are shown in Figure 5(c), revealing the dispersion relationship of *E*_UPB_ and *E*_LPB_. At the emission angle of 24°, the Rabi splitting was calculated as $\hbar \Omega_{\text{Rabi}}=2\sqrt{{g_{A}}^{2}}=45\;meV$ , satisfying the strong coupling condition *ℏ*Ω_Rabi_ > (*ℏ*Γ_*ex*_ + *ℏ*Γ_MC_)/2. This confirms the system is in the strong coupling regime. Figure 5(d) and (e) show the exciton and photon components of the polariton states, described by weighing fractions calculated as Hopfield coefficients, plotted as functions of cavity detuning [90].

## 4 Conclusion

In this study, we synthesized a CsPbBr_3_@UiO-66 composite by assembling CsPbBr_3_ QDs within UiO-66 micropores. This approach enhanced the environmental stability of CsPbBr_3_ QDs, enabling continuous luminescence for over 30 months in ambient air and stable emission in water for several days. This stability addresses a critical limitation of traditional perovskite materials in optoelectronic applications. Temperature-dependent PL and TRPL measurements revealed detailed exciton–phonon interactions, providing insights into the material’s fundamental optical properties. We anticipate that these optical features can be generalized to other MOFs and perovskite QD compositions. We anticipate that these optical features can be generalized to other MOFs and perovskite QD compositions. With the porous MOF to coordinate metal ions such as Pb^2+^ first, followed by the heterogeneous reaction between the porous solid and another precursor to form the perovskite QDs confined within the MOF pore, the size and optical property of the resulting perovskite QDs should be highly tunable by simply adjusting the pore size and pore structure of MOFs. However, since most MOFs themselves are not chemically stable in water [91], the careful selection of MOFs that are both stable in water as well as capable of coordinating Pb^2+^ ions prior to the further formation of perovskite is necessary. In addition to UiO-66, such a two-step formation of pore-confined perovskite QDs should also be generalizable to other group (IV) metal-based MOFs such as MOF-808, NU-1000 and PCN-222, which are all chemically robust in water and possess terminal –OH/OH_2_ pairs on their nodes for the installation of guest metal ions by SIM [92]. Taken together, these findings suggest that the rational selection of MOFs – featuring chemical stability, pore size compatibility, and low background emission – could provide a universal strategy for enhancing the optical performance and durability of perovskite QDs. The strong light–matter interaction in hybrid MC demonstrated the formation of hybrid polaritonic states, positioning the CsPbBr_3_@UiO-66 composite as a promising candidate for polaritonic devices and strong coupling studies. The combination of superior environmental stability and strong coupling behavior highlights the CsPbBr_3_@UiO-66 composite as a robust and versatile platform for advanced optoelectronic applications. These results advance perovskite-based technologies in polaritonics [76], low-threshold lasing [77], and emerging photonic applications [78], [79], [93]. Future work could optimize optical properties and explore scalable integration.

## Supplementary Material

Supplementary Material Details
