# Supplementary material for: Achieving long-term water stability and strong exciton–photon coupling in CsPbBr3 quantum dots via MOF encapsulation
Source: Nanophotonics. 2025 Jun 18;14(14):2397–409. doi: 10.1515/nanoph-2025-0059 (PMC12273544; doi:10.1515/nanoph-2025-0059)
Supplement: Supplementary file 1 — Supplementary Material Details [file j_nanoph-2025-0059_suppl_001.docx]

**Supporting information**

**Title**

Achieving Long-Term Water Stability and Strong Exciton–Photon Coupling in CsPbBr_3_ Quantum Dots via MOF Encapsulation

AUTHORS

Chiao-Chih Lin^1^, Shih-Cheng Wan^2^, Cheng-Hui Shen^3^, Zheng-Lin Liao^2^, Yen Liu^2^, Zong Yu Wu^2^, Sheng-Chan Wu^2^, Chia-Kai Lin^2^, Chung-Wei Kung^3^, Hsu-Cheng Hsu^1,2,4^, Yu-Hsun Chou^1,2,4^

^1^ Program on Key Materials, Academy of Innovative Semiconductor and Sustainable Manufacturing, National Cheng Kung University, Tainan 701, Taiwan

^2^ Department of Photonics, National Cheng Kung University, Tainan 701, Taiwan

^3^ Department of Chemical Engineering, National Cheng Kung University, Tainan, 70101 Taiwan

^4^ Meta-nanoPhotonics Center, National Cheng Kung University, Tainan 701, Taiwan

**Synthesis of CsPbBr_3_@UiO-66**

Add 43 μL of zirconium (IV) propoxide solution (70 wt% in 1-propanol, Sigma-Aldrich), 4.2 mL of dimethylformamide (DMF, >99.8%, ECHO Chemical Co, Ltd, Taiwan), and 2.4 mL of acetic acid (>99.8%, Honeywell Fluka) into a glass vial. Heat the mixture in an oven at 130 °C for 2 hours. After heating, remove the vial and cool it down to room temperature. Once cooled, add 49 mg of terephthalic acid (H_2_BDC, 98%, Sigma-Aldrich) to the vial and stir at room temperature by using a magnetic stir bar at 1000 rpm for 20 hours. After stirring, add 10 mL of DMF and use centrifuge to remove excess reactants. Repeat this step three times. Next, perform a solvent exchange by adding 10 mL of acetone (>99.0%, ECHO Chemical Co, Ltd, Taiwan). Centrifuge and repeat this step three times, soaking the sample in acetone for 2 hours, overnight, and another 2 hours before each centrifugation. Finally, dry the solution in an oven at 80 °C to obtain defective UiO-66 powder. Place 56.6 mg of PbBr_2_ (99.998%, Alfa Aesar), 30 mg of defective UiO-66 powder, and 6.17 mL of DMF into a glass vial, and stir the mixture at 600 rpm with a magnetic stir bar at room temperature for 24 hours. After stirring, add 10 mL of DMF, centrifuge to remove excess reactants, and repeat this step three times. Next, add 10 mL of acetone for solvent exchange, and similarly, centrifuge and repeat this step three times, soaking the sample in acetone for 2 hours, overnight, and another 2 hours before each centrifugation. Finally, dry the solution in an oven to obtain Pb-UiO-66 powder. Add 42.5 mg of CsBr powder (99.999%, Alfa Aesar), 9.9 mL of methanol (>99.9%, Tedia), and 0.1 mL of deionized water into a glass vial to prepare a 0.02 M CsBr precursor solution. Deionized water was used as the water source throughout this work. Once the CsBr powder is fully dissolved, take 2 mL of the CsBr precursor solution and mix it with 19.22 mg of Pb-UiO-66 powder. After mixing, place the sample vial in an ultrasonic bath for 10 minutes to ensure the CsBr precursor solution is evenly diffused into the pores of the Pb-UiO-66 powder. The CsBr precursor solution disrupts the bonds between lead and oxygen atoms in the Pb-UiO-66 powder, leading to the formation of CsPbBr_3_ perovskite QDs at the lead ion sites. Finally, the mixture was stirred at 600 rpm on a 90 °C heating plate and dried in air. The resulting solid was collected and designated as “CsPbBr_3_@UiO-66”.

**Synthesis of CsPbBr_3_ QDs and Thin film (TF)**

The detail of CsPbBr_3_ QDs used in this study can be refer to this aritcle.^1^ For the synthesis of CsPbBr₃ thin films, a precursor solution was prepared by dissolving CsBr (26.6 mg) and PbBr₂ (45.9 mg) in a mixed solvent of dimethyl sulfoxide (DMSO, 0.4 mL) and gamma-butyrolactone (GBL, 0.1 mL) at a volume ratio of 4:1. All materials are purchase from Sigma Aldrich, USA. The resulting solution, with a final molar concentration of 0.25 M, was heated at 60°C to facilitate complete dissolution of the precursors. The mixed precursor solution was spin-coated onto a substrate using a single-step acceleration method. The spin-coating process was conducted at a rotation speed of 4500 rpm for 30 seconds. After spin-coating, the substrate was transferred to a hot plate and annealed at 210°C for 5 minutes

**Instrumentation**

The morphology and elemental composition of the CsPbBr_3_@UiO-66 composite were analyzed using a scanning electron microscope (SEM, Hitachi, SU-8010) equipped with an energy-dispersive X-ray spectroscopy (EDX) system. Transmission Electron Microscopy (TEM) was conducted using a JEM-2800F system, with the measurements provided by Integrated Service Technology. The crystal structure of the composite was determined via X-ray diffraction (XRD) using a Rigaku Ultima IV powder diffractometer with CuKα radiation. Nitrogen adsorption-desorption isotherms were measured at 77 K using a Micromeritics ASAP 2020 system to evaluate the surface area and porosity characteristics. The surface area was calculated using the Brunauer-Emmett-Teller (BET) method based on the obtained isotherms. The pore size distribution was estimated by applying density functional theory (DFT) with the carbon slit-pore model, also utilizing the obtained isotherms. Fourier-transform infrared spectroscopy (FTIR) data were collected by using a Nicolet 6700 (Thermo Fisher Scientific). The accelerated aging test is done by using a HRMB-80 Test Chamber (Terchy Environmental Technology Ltd).

**Optical Measurement details**

*TRPL measurement:* A diode-pumped passively Q-switched laser centered at 355 nm with pulse duration of 350 ps and repetition rate of 1 kHz was used to excite the sample.

*Stability measurement:* A 405 nm diode CW laser was used to excite the sample with a focused beam size of ≈1.6 µm in diameter.

*ARPL* *measurement:* Emissions from the device were pumped by 325 nm He-Cd laser, collected along the same path and directed into a spectrometer (Horiba-Jobin-Yvon iHR320) with a liquid-nitrogen-cooled CCD array detector. The spectral resolution of the spectrometer with a 1800 lines/mm grating is 0.29 nm.

*Low-temperature measurements:* CsPbBr_3_@UiO-66 was placed in a cryostat (Montana Instrument) pumped by a mode-locked Ti:sapphire regenerative amplifier (Verdi G6, Coherent, America) with 35-fs pulse duration and 1 kHz repetition rate at 405 nm.

**Accelerated aging experiments**

CsPbBr_3_@UiO-66 was placed in a chamber maintained at 65°C and 65% relative humidity. As shown in Figure S1, even after 100 hours, the luminescence intensity remains essentially unchanged compared to the initial value, demonstrating its effective ability to block the influence of moisture.

|  |
| --- |
| Figure S1. CsPbBr_3_@UiO-66 accelerated aging test. |

**Interplanar Spacing and Lattice Structure**

Table S1 presents the calculated interplanar spacings for the (hkl) planes of CsPbBr_3_@UiO-66 derived from XRD analysis. These values are compared with the interplanar spacings from the lattice fringes in the TEM micrographs. The observed fringes show excellent agreement with the calculated values. This consistency confirms the phase and crystallinity of CsPbBr_3_@UiO-66, further supporting the successful synthesis of the CsPbBr_3_ within the UiO-66 framework.

**Table S1** Comparison of the interplanar spacing between XRD and TEM.

| Plane (hkl) | Interplanar spacing (Å) from XRD | Interplanar spacing (Å) from TEM |
| --- | --- | --- |
| (220) | 3.373 | 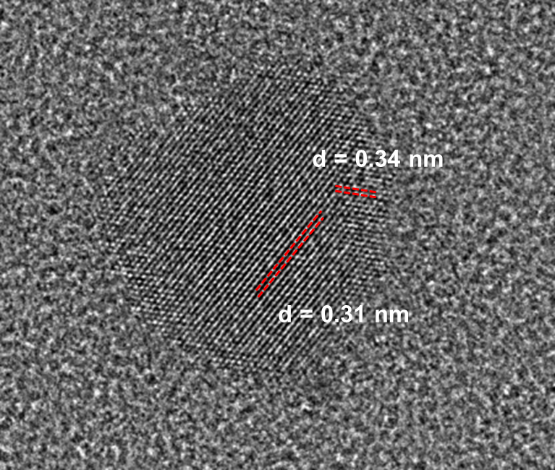 |
| (221) | 3.117 |  |
| (131) | 3.245 | 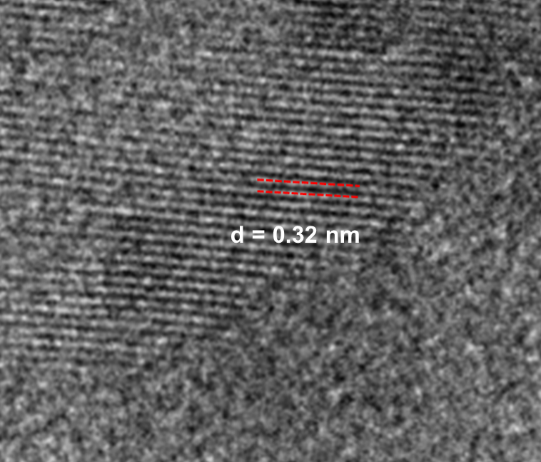 |
| (202) | 2.906 | 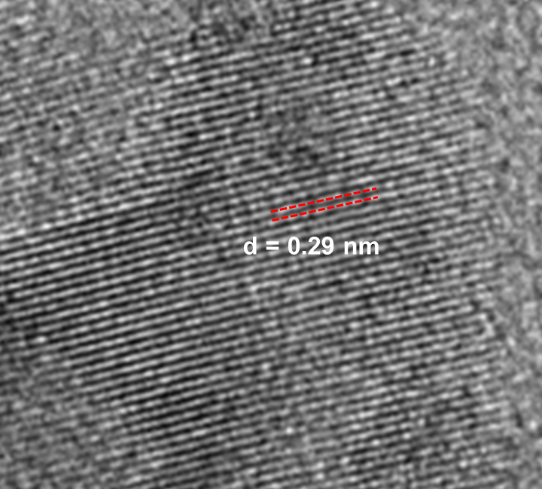 |

| 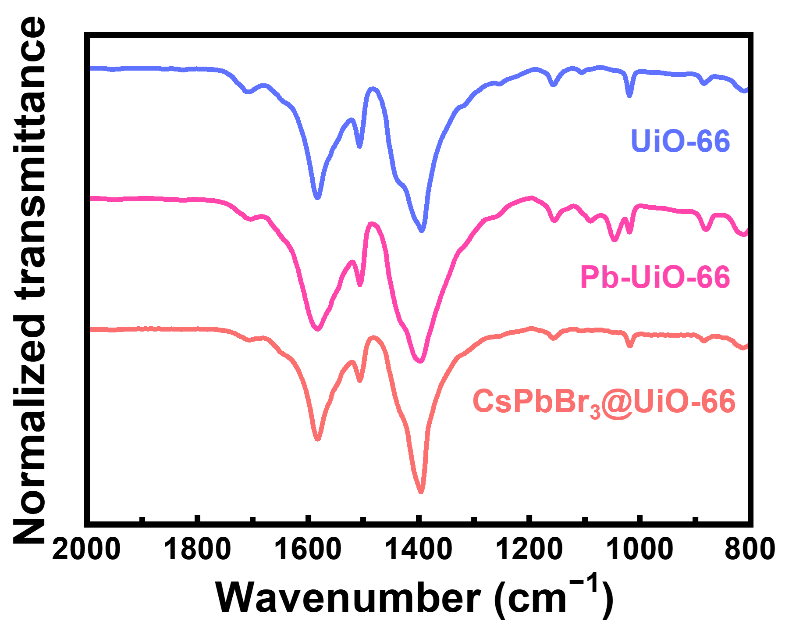 |
| --- |
| Figure S2. FTIR spectra of UiO-66, Pb-UiO-66, and CsPbBr_3_@UiO-66 |

**Table S2**. Summary of the fitting results corresponding to the plot of PL spectrum

| Equation | $I\left( T \right)= \frac{I_{0}}{1+Ae^{-\frac{E_{b}}{k_{B}T}}}$ |
| --- | --- |
| Adjusted r^2^ | 0.992 |

| Parameter | *A* (a.u.) | $E_{b} (meV)$ |
| --- | --- | --- |
| Value | 11.9212 | 59.0 ± 0.8 |

**Table S3**. Summary of the fitting results corresponding to the plot of PL peak energy

| Equation | $E_{g}\left( T \right)=E_{g,0}+C_{th}\cdot T+C_{ep}\left( 1+\frac{2}{e^{\frac{\hbar\omega}{k_{B}T}}-1} \right)$ |
| --- | --- |
| Adjusted r^2^ | 0.982 |

| Parameter | $E_{g,0}(eV)$ | $C_{th}(eV/K)$ | $C_{ep}(meV)$ | $\hbar\omega(meV)$ |
| --- | --- | --- | --- | --- |
| Value | 2.367 | $\text{8.1×}\text{10}^{\text{-5}}$ | 31.7 ± 3 | 47.0 ± 5 |

**Table S4**. Summary of the fitting results corresponding to the plot of PL linewidth

| Equation | $\Gamma\left( T \right)= \Gamma_{0}+\frac{\Gamma_{op}}{e^{(\frac{\hbar\omega_{op}}{k_{B}T})}-1}$ |
| --- | --- |
| Adjusted r^2^ | 0.990 |

| Parameter | $\Gamma_{0} (meV)$ | $\Gamma_{op}(meV)$ | $\hbar\omega_{op} (meV)$ |
| --- | --- | --- | --- |
| Value | 32.87 | 90.57 | 29 ± 3 |

| **** |
| --- |
| **Figure S3**. Linewidth of the PL spectrum. The solid line is the fit according to equation (3). |

**TRPL Fitting**

Table S5 shows the parameters of the TRPL fitting curve and Figure S4 displays the decay profile of the materials, where the experimental data are represented by scattered points, and the corresponding fitting curves are illustrated by solid lines. The fitting was carried out using a double-exponential decay function: $I(t)= A_{1}e^{\frac{-t}{\tau_{r}}}+A_{2}e^{\frac{-t}{\tau_{nr}}}$. , The individual radiative ($\tau_{r}$) and non-radiative lifetimes ($\tau_{nr}$) components are clearly shown as blue and red solid lines, respectively. The fitting curves demonstrate good agreement with the experimental data, validating the reliability of the extracted lifetime parameters listed in Table S5.

**Table S5**. Kinetic parameters of the TRPL spectrum of different materials

| Material | *A_1_* | $\tau_{r} (ns)$ | *A_2_* | $\tau_{nr} (ns)$ | $\tau_{avg} (ns)$ |
| --- | --- | --- | --- | --- | --- |
| QD | 0.801 | 1.34 | 0.199 | 4.04 | 2.50 |
| CsPbBr_3_@UiO-66 | 0.780 | 3.00 | 0.228 | 16.52 | 11.34 |
| TF | 0.460 | 6.35 | 0.591 | 15.21 | 13.04 |

| **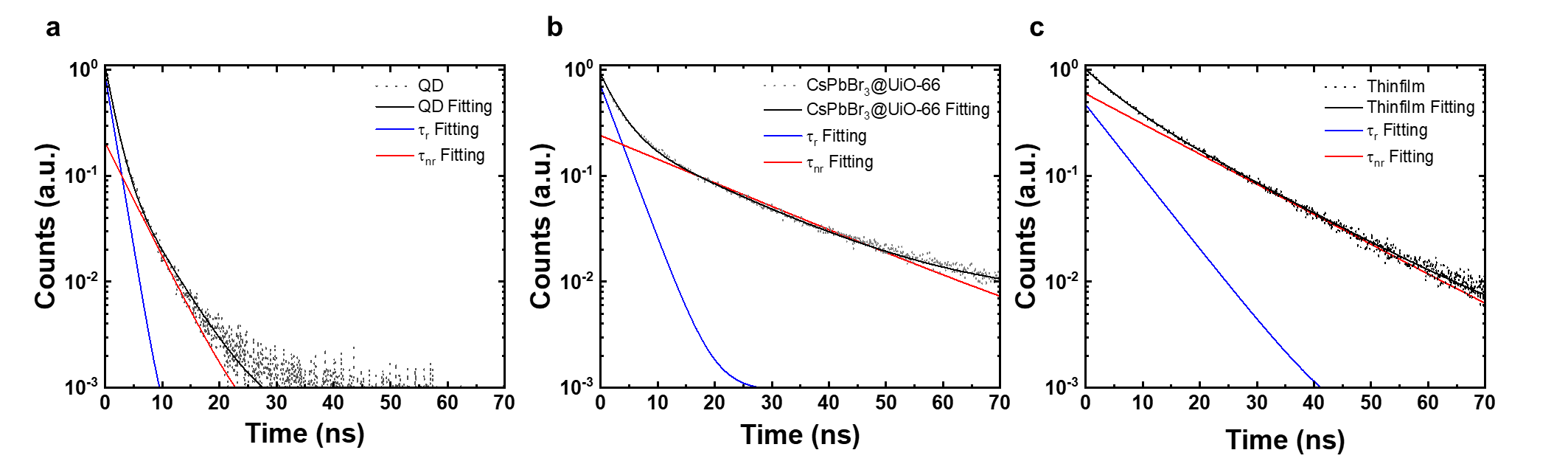** |
| --- |
| **Figure S4.** TRPL decay curve of a) QD b) CsPbBr_3_@UiO-66 and c) TF, showing radiative $\tau_{r}$ (blue), $\tau_{nr}$ (red) and overall double-exponential fitting (black). Gray dots represent experimental data. |

**Trap States Density Determination**

In the study of defect states in CsPbBr_3_ materials, it is often classifying trap states into two main categories: bulk traps and surface/interface traps [1], [2], [3], [4]. This classification is widely used in both experimental and theoretical analyses due to its clarity and ability to explain most observed phenomena. In the case of CsPbBr_3_@UiO-66, the presence of the UiO-66 framework introduces additional interface roughness and defects, which predominantly contribute to surface traps. However, TRPL measurements have shown that the non-radiative recombination lifetime of CsPbBr_3_@UiO-66 is significantly longer than that of thin films. This suggests that while UiO-66 may introduce surface/interface traps, it also reduces the impact of bulk traps by passivating intrinsic defects within the quantum dots. Therefore, we focus on discussing these two trap types and we compare CsPbBr_3_@UiO-66 with QDs and thin film (TF). We assumed that under low fluence fs laser pulse excitation, the band-edge radiative recombination is much faster than the recombination trap states. The dynamics of photo-generated charge carrier density $n_{C}\left( t \right)$ can be described by the following set of differential equations.

$\frac{dn_{C}(t)}{dt}=-a_{bulk}n_{C}\left( t \right)n_{T}^{bulk}\left( t \right)-a_{surf}n_{C}\left( t \right)n_{T}^{surf}\left( t \right)-\frac{n_{C}(t)}{\tau_{0}}$ (4.1)

$\frac{dn_{T}^{bulk}(t)}{dt}=-a_{bulk}n_{C}\left( t \right)n_{T}^{bulk}\left( t \right)$ (4.2)

$\frac{dn_{T}^{surf}(t)}{dt}=-a_{surf}n_{C}\left( t \right)n_{T}^{surf}\left( t \right)$ (4.3)

where the term $n_{T}^{bulk}\left( t \right)$ and $n_{T}^{surf}\left( t \right)$refers to the density of bulk and surface trap states, while $a_{i}$ is the product of trapping cross-section and the carrier velocity. In Equation (4.1), the first and second product represent trap-mediated non-radiative pathways, the third term denotes the radiative recombination inside the material. The relationship between initial photogenerated charge carrier density $n_{C}\left( 0 \right)$ and integrated PL intensity ($I_{PL}$)can be derived as:

$n_{C}\left( 0 \right)=n_{T}^{bulk}\left( 0 \right)\left( 1+e^{\frac{-a_{bulk}\tau_{0}I_{PL}}{k}} \right)+n_{T}^{surf}\left( 0 \right)\left( 1+e^{\frac{-a_{surf}\tau_{0}I_{PL}}{k}} \right)+\frac{I_{PL}}{k}$ (4.4)

where k is a fitting constant. Fitting the experimental results with equation (4.4), we plotted the PL intensity values of CsPbBr_3_@UiO-66, QDs, and TF against the fitted curves. The results demonstrate an excellent agreement between the experimentally obtained PL data points and the fitted curves, as shown in Figure S5. The details of the fitting parameters are in Table S6. This correlation validates the accuracy of the model in estimating the total trap density (including bulk and surface traps) for these materials. Through Figure S5, at low pump fluence, the majority of photogenerated carriers are captured by trap states, including bulk traps and surface/interface traps. As the pump fluence increases, the trap states are progressively filled. Once the trap states become saturated, radiative recombination starts to dominate, leading to a significant increase in PL intensity, as observed in the experimental results for QDs (square dots) and the fitting curve (blue line). Similarly, in TF, we can observe comparable behavior (orange stars, orange line). As for CsPbBr_3_@UiO-66, the PL intensity exhibits a linear increase with increasing pump fluence. We attribute this phenomenon to the influence of surface trap states. Due to its porous structure and extensive surface area, the UiO-66 framework introduces many surface/interface trap states. These traps dominate the carrier dynamics by capturing photogenerated carriers and suppressing radiative recombination. Although the UiO-66 framework effectively passivates the bulk traps in CsPbBr_3_@UiO-66, it makes the carrier dynamics more influenced by surface traps.

These numerous surface traps are uniformly distributed throughout the material, ensuring that even at higher pump fluence levels, a significant number of carriers are still captured by these traps. Thereby suppressing a substantial increase in PL intensity.

| 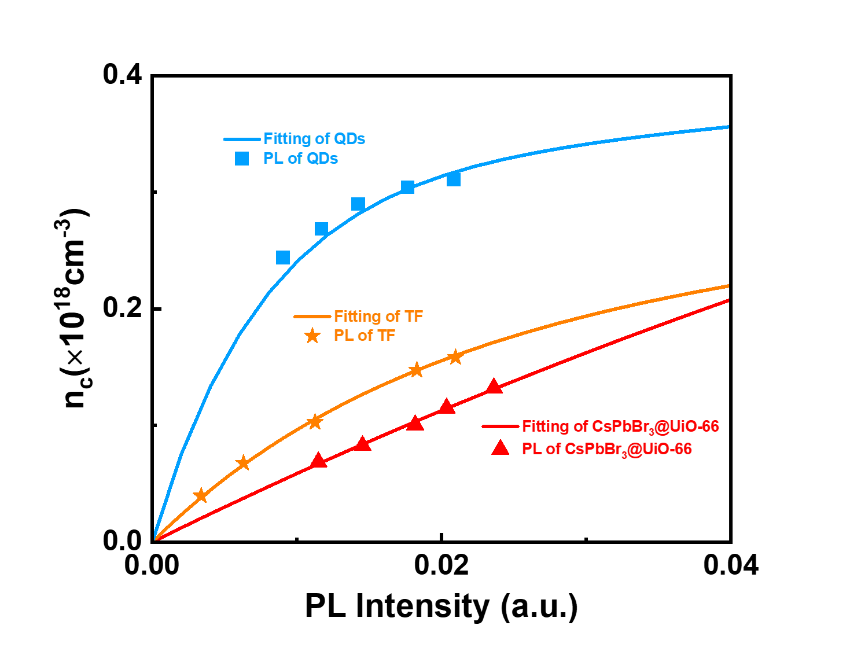 |
| --- |
| **Figure S5.** Determination of the trap state densities. PL intensity as a function  of photon-generated exciton density within the low pump fluence range. |

**Table S6** Summary of the fitting results corresponding to equation (4.4) and Figure S5.

|  | QDs | CsPbBr_3_@UiO-66 | TF |
| --- | --- | --- | --- |
| $n_{T}^{bulk}\left( 0 \right)$ | $1\times{10}^{15}$ | $1\times{10}^{14}$ | $2\times{10}^{16}$ |
| $n_{T}^{surf}\left( 0 \right)$ | $3.1\times{10}^{17}$ | $6.4\times{10}^{18}$ | $2\times{10}^{17}$ |

**Compare the Absorption of CsPbBr_3_@UiO-66, QDs and TF**

We measure the absorption spectrum at room temperature, and model that in the framework of the Elliot’s theory including hydrogen-like excitonic effects [5], [6], [7]. The excitonic absorption spectrum ($\alpha_{ex}$) is modeled as a combination of discrete states and continuum states since it exhibits both quantized bound states and unbound states above the bandgap energy:

$\alpha_{ex}=\left[ A\Theta\left( E-E_{g} \right)\cdot D_{CV}\left( E \right) \right]\cdot\frac{\pi x\left( E \right)\cdot\exp\left( \pi x\left( E \right) \right)}{\sinh\left( \pi x\left( E \right) \right)}+AE_{b}\sum_{i}^{\infty} \frac{4\pi}{n^{3}}\cdot\delta\left( E-E_{g}+\frac{E_{b}}{n^{2}} \right)$ (5.1)

where A is a constant related to the transition matrix element, $E$ is the photon energy, $E_{g}$ is the bandgap, $\Theta$ is the Heaviside step function. $D_{CV}\left( E \right)$ is the is the joint density of valence and conduction bands states, $\delta$ is the delta function, $E_{b}$ is the exciton binding energy and $x$ is defined as:

$$x=\sqrt{E_{b}\cdot(E-E_{g})}$$

| 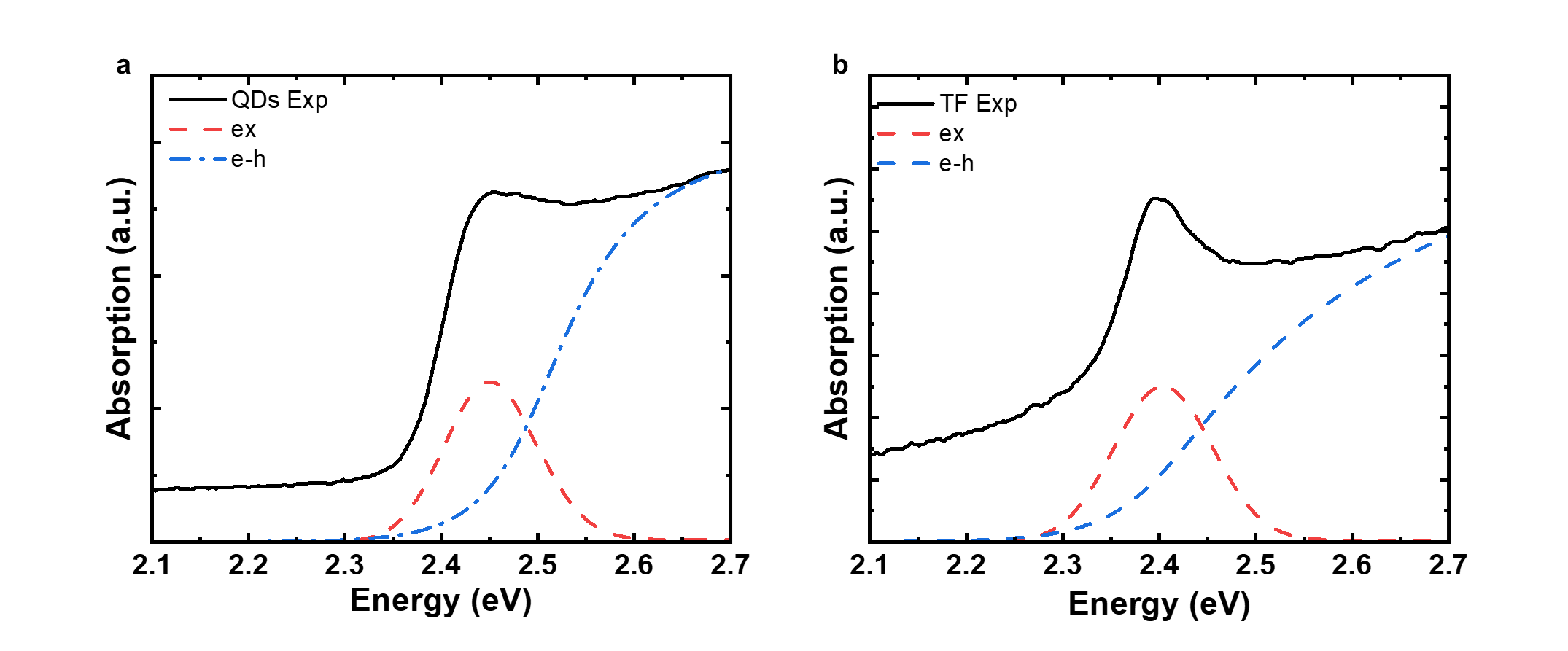 |
| --- |
| **Figure S6.** Absorption spectrum of the a) CsPbBr_3_ QDs and b) TF. The black line shows the experimental data, along with the theoretical fit of contributions of the excitonic (red dashed line) and the band-to-band (blue dashed line) transitions are estimated according to Elliot’s theory of Wannier excitons. |

**Table S7** Summary of the fitting results corresponding to equation (5.1) and Figure S6 and compare with Figure 3(e).

| Material | QDs | CsPbBr_3_@UiO-66 | TF |
| --- | --- | --- | --- |
| $E_{b}$ (meV) | 45±5 | 60±3 | 30±4 |

**Device Fabrication**

The DBR is designed for a center wavelength of 528 nm, using SiO_2_ and TiO_2_. The refractive index of SiO_2_ is set to 1.5 and TiO_2_ to 2.5. We use λ/4n to define the layer thickness of the DBR. The DBR was composed of 9 layers of TiO_2_/SiO_2_ on a Si substrate by the electron beam evaporation process. We prepared the spin-coating solution by adding the synthesized CsPbBr_3_@UiO-66 powder to a glass vial at a ratio of 3 mg per 1 mL of dichloromethane (DCM, 99.98%, Sigma–Aldric) solution. The vial was then placed in an ultrasonic bath for at least 30 minutes to ensure complete dispersion of the CsPbBr_3_@UiO-66 powder, forming a uniform suspension. Subsequently, 0.1 mL of the spin-coating solution was dropped onto the center of the bottom DBR. Spin-coating was conducted in two stages: the first stage at 2500 rpm for 30 seconds, followed by a second stage at 4000 rpm for 5 seconds. Then, TiO_2_ layer was deposited on top of the CsPbBr_3_@UiO-66. Finally, a 60 nm-thick Ag layer was deposited on the top of it. Figure S7(a) shows the electric ﬁeld distribution in which the strong electric ﬁeld conﬁnement can be clearly observed at the exciton emission wavelength. The ﬁnite element method, commercial software (COMSOL Multiphysics) was used to calculate the electric ﬁeld distribution. In this modeling, we adopted 2D structures in the models where the periodic boundary conditions are used [8]. The electric ﬁeld distribution (|E|) was extracted along the structure. Figure S7(b) shows the experimental results for reﬂectance spectra of the device cavity mode (black line) measured by UV/Vis/NIR spectroscopy (HITACHI U4100) and the emission spectra of excitons of CsPbBr_3_@UiO-66 are investigated by PL spectroscopy (blue line).

| 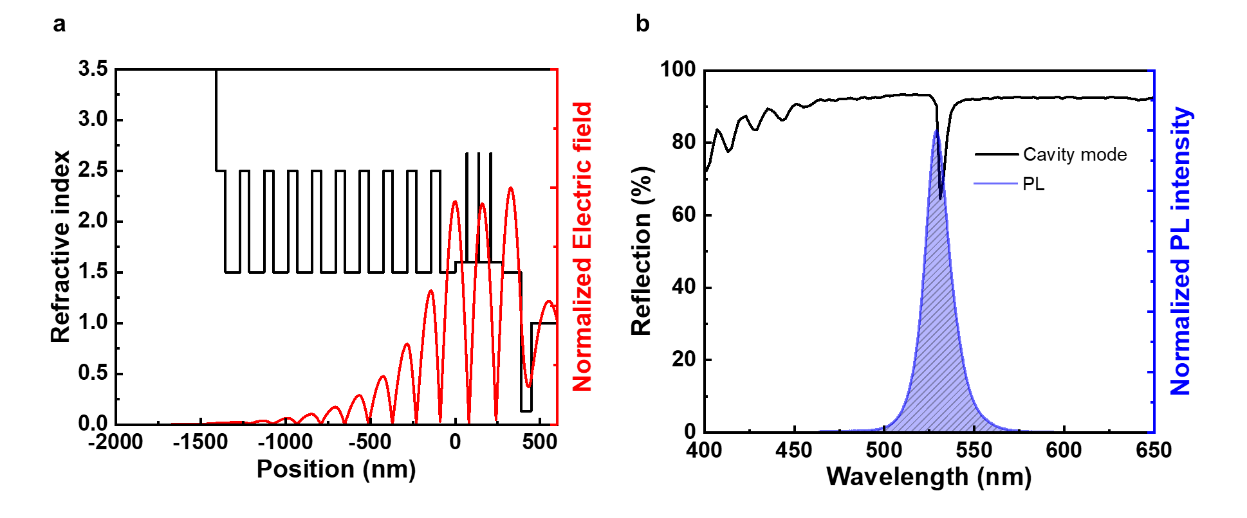 |
| --- |
| **Figure S7.** a) The electric field distribution at wavelength = 528 nm, and the refractive index profile (from left to right: Si substrate, device and air) for the proposed device structure. Since multi-CsPbBr_3_@UiO-66 may be excited at the same time, we set the it as a kind of quantum well structure. b) The optical properties of CsPbBr_3_@UiO-66 were conﬁrmed by PL measurement (blue area) and the reﬂectance spectra of the cold cavity (black line). |

**References**

[1] B. A. Koscher, J. K. Swabeck, N. D. Bronstein, and A. P. Alivisatos, “Essentially Trap-Free CsPbBr3 Colloidal Nanocrystals by Postsynthetic Thiocyanate Surface Treatment,” *J. Am. Chem. Soc.*, vol. 139, no. 19, pp. 6566–6569, May 2017, doi: 10.1021/jacs.7b02817.

[2] K. Intonti *et al.*, “Role of interface and bulk traps on the capacitance–voltage characteristics of WS2/Al2O3/Si capacitors,” *Solid-State Electronics*, vol. 207, p. 108697, Sep. 2023, doi: 10.1016/j.sse.2023.108697.

[3] X. Jin *et al.*, “Trap states engineering toward all-inorganic CsPbBr3 perovskite nanocrystals for highly efficient light-emitting diodes,” *J. Mater. Chem. C*, vol. 11, no. 29, pp. 9996–10003, Jul. 2023, doi: 10.1039/D3TC00702B.

[4] Y. Zhao *et al.*, “Surface passivation of CsPbBr3 films by interface engineering in efficient and stable self-powered perovskite photodetector,” *Journal of Alloys and Compounds*, vol. 965, p. 171434, Nov. 2023, doi: 10.1016/j.jallcom.2023.171434.

[5] M. Saba *et al.*, “Correlated electron–hole plasma in organometal perovskites,” *Nature Communications*, vol. 5, no. 1, p. 5049, Sep. 2014, doi: 10.1038/ncomms6049.

[6] M. Baranowski and P. Plochocka, “Excitons in Metal-Halide Perovskites,” *Advanced Energy Materials*, vol. 10, no. 26, p. 1903659, 2020, doi: 10.1002/aenm.201903659.

[7] M.-C. Yen *et al.*, “Tamm-Plasmon Exciton-Polaritons in Single-Monolayered CsPbBr3 Quantum Dots at Room Temperature,” *Advanced Optical Materials*, vol. 11, no. 4, p. 2202326, 2023, doi: 10.1002/adom.202202326.

[8] W.-H. Xu *et al.*, “Tamm Plasmon-Polariton Ultraviolet Lasers,” *Advanced Photonics Research*, vol. 3, no. 1, p. 2100120, 2022, doi: 10.1002/adpr.202100120.
